# Global elevation of algal bloom frequency in large lakes over the past two decades

**DOI:** 10.1093/nsr/nwaf011

**Published:** 2025-01-11

**Authors:** Ying Wang, Dan Zhao, R Iestyn Woolway, Haoran Yan, Hans W Paerl, Yi Zheng, Chunmiao Zheng, Lian Feng

**Affiliations:** School of Environmental Science and Engineering, College of Engineering, Southern University of Science and Technology, Shenzhen 518055, China; School of Environmental Science and Engineering, College of Engineering, Southern University of Science and Technology, Shenzhen 518055, China; School of Ocean Sciences, Bangor University, Menai Bridge, LL59 5AB, UK; Faculty of Resources and Environmental Science, Hubei University, Wuhan 430062, China; Institute of Marine Sciences, University of North Carolina at Chapel Hill, Morehead City, NC 28577, USA; School of Environmental Science and Engineering, College of Engineering, Southern University of Science and Technology, Shenzhen 518055, China; Eastern Institute for Advanced Study, Eastern Institute of Technology, Ningbo 315200, China; School of Environmental Science and Engineering, College of Engineering, Southern University of Science and Technology, Shenzhen 518055, China

**Keywords:** lakes, algal blooms, satellite observations, climate warming

## Abstract

The recent increase in algal blooms in lakes, potentially exacerbated by climate warming, is of global concern. However, a spatially and temporally detailed characterization of algal bloom trends at a global scale has been lacking, posing challenges to definitively attribute the increase trend to warming as a primary driver. Here, we used daily MODIS satellite observations from 2003 to 2022 to analyze algal bloom trends in 1956 large freshwater lakes worldwide. Among these lakes, 620 have experienced algal bloom events in over half of the years during the past two decades, with an upward trend in bloom frequency observed in 504 lakes. This trend is particularly prominent in subtropical regions and has become most pronounced after 2015. The global median annual bloom frequency has significantly increased at a rate of +1.8%/yr over the past two decades, showing a significant correlation with air temperatures (*r*^2^ = 0.43, *P* < 0.05). Furthermore, in 44.8% of the bloom-affected lakes, we observed a strong correlation between air temperature and bloom frequency. Our study helps clarify the factors contributing to the global expansion of algal blooms and emphasizes the urgent need to recognize and address this growing environmental challenge within the context of climate warming.

## Teaser

Algal blooms in lakes have increased globally, with a marked upward trend observed in 504 of 1,956 large freshwater lakes analyzed using MODIS satellite data from 2003 to 2022. This trend, especially pronounced in subtropical regions since 2015, is closely correlated with air temperature in 44.8% of bloom-affected lakes, underscoring the influence of climate warming on this escalating environmental issue.

## INTRODUCTION

The proliferation of harmful algal blooms in both inland and coastal waters constitutes a critical global environmental and public health concern [1,2]. Over the past four decades, freshwater algal blooms have been documented in more than 20 000 lakes worldwide, encompassing ∼57% of the total global lake surface area [2]. Algal blooms in freshwater ecosystems can cause severe ecological, societal and economic consequences. These include disruptions to drinking water supplies, incidents of critical fish and pet mortality, and a reduction in tourism, among other concerns [3]. It is estimated that in China alone, the economic losses linked to harmful algal blooms amount to ∼6.5 billion US dollars annually [4].

The global proliferation of algal blooms has been extensively documented since the 1960s [5], primarily attributed to the increase in nutrient pollution stemming from sources such as detergents and fertilizers [6]. In response to these concerns, numerous local governments launched substantial water quality management initiatives around the 1970s [7–9]. These efforts have yielded some measures of success in mitigating algal blooms [10]. However, from the late 20th century to present, there has been a resurgence of algal blooms in lakes that were previously rehabilitated, despite the fact that the nutrient input levels in these lakes have not shown a corresponding increase [11,12]. Moreover, there has been a widespread increase in the number of lacustrine algal blooms worldwide, with numerous lakes now experiencing blooms on an annual basis [2,13,14].

Recent studies have sought to link the global increase in lake algal blooms to climate warming [15–18], with strong evidence from both laboratory culture experiments and ecosystem observations suggesting a positive feedback between rising temperatures and algal blooms [19,20]. Warming temperatures are often associated with accelerated phytoplankton rates [21], enhanced stratification [22] and an extended growing season [13]—all conditions that favor bloom development. Yet, a fundamental question remains unanswered: To what extent is climate warming responsible for the recent increase in lake blooms at the global scale?

Addressing the above question presents a substantial challenge due to the absence of a comprehensive spatial and temporal characterization of global algal bloom trends. While satellite remote sensing provides valuable synoptic global observations [23–25], the most comprehensive dataset available concerning global lake algal blooms is currently limited to decadal mean changes due to inadequate observational frequency [2]. Despite prior efforts that have utilized high-frequency satellite data to investigate the expansion of algal bloom extent and duration, these endeavors have typically been confined to individual lakes or regional studies [26,27]. To address this knowledge gap, our study utilized frequent observations spanning the globe from 2003 to 2022 to comprehensively characterize algal blooms in large lakes, shedding light on the potential interplay between climate warming and these trends.

## RESULTS AND DISCUSSION

### Daily mapping of global lake algal blooms

**Figure 1.**
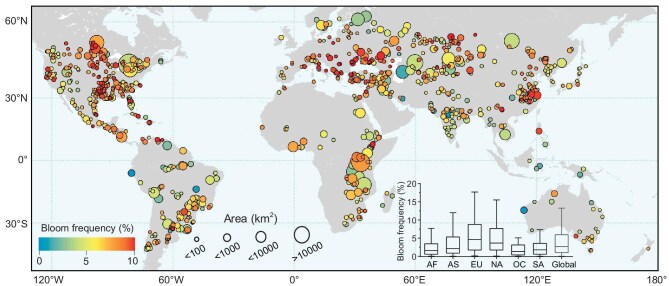
Global patterns of algal blooms in large freshwater lakes between 2003 and 2022. The 20-year mean annual bloom frequency for bloom-affected lakes (*n* = 962) is color-coded. Boxplots show the global and continental statistics, where the center line represents the median value, the bottom and top bounds of boxes are first and third quartiles, and the whiskers show a maximum of 1.5 times the interquartile range. South America (SA), *n* = 117; Africa (AF), *n* = 88; Europe (EU), *n* = 139; North America (NA), *n* = 279; Asia (AS), *n* = 316; Oceania (OC), *n* = 24.

We conducted a comprehensive analysis of algal blooms in large (>50 km^2^) freshwater lakes and reservoirs (collectively referred to here as ‘lakes’) worldwide utilizing daily observations from Moderate Resolution Imaging Spectroradiometer (MODIS) satellite images. Our analysis included 0.8 million images captured between 2003 and 2022, utilizing a recently developed algal bloom detection algorithm (uncertainty <20%) (see Methods) [28]. Our study covered 1956 lakes distributed across the globe (Fig. 1), which together represent 51% of the total surface area of freshwater lakes worldwide [29–31]. An algal bloom is here defined as the surface accumulation of phytoplankton that exhibits a detectable signal from satellite observations. This definition does not provide information on subsurface blooms, phytoplankton species composition and toxicity, or the exact value of phytoplankton biomass [32,33].

This extensive dataset enabled us to investigate key parameters associated with lake algal blooms, including their frequency, onset and persistence (see Methods) (Fig. S1). Specifically, we quantified bloom frequency as the proportion of the bloom area relative to the satellite observational area. In the Northern Hemisphere, bloom onset was defined as the first day of the year when blooms were detected, while bloom persistence was defined as the duration between the initial and final bloom observations within a given year. For the Southern Hemisphere, these metrics were adjusted to account for hemispherical seasonal differences (see Methods). For those lakes in which blooms were identified during the past two decades, we calculated the annual values for these three parameters. We further estimated their rate of change through linear regression slopes, with a particular focus on lakes where blooms occurred in more than half of the observational record.

### Global bloom patterns

Algal blooms were detected in approximately half (49.2% or 962) of the large lakes under examination, hereafter referred to as ‘bloom-affected lakes’. Of these, 585 lakes have experienced annual blooms consistently over the past 20 years (Fig. 1). The long-term bloom frequency for these bloom-affected lakes exhibited a median annual value of 2.2%. Algal blooms were prevalent on all six continents, with subtropical lakes (median annual bloom frequency = 2.2%, *n* = 387) experiencing more frequent blooms compared to tropical lakes (median annual bloom frequency = 1.0%, *n* = 200). Additionally, lakes with a surface area of <1000 km^2^ exhibited a higher bloom frequency (3.3%) compared to larger lakes (2.3%). This difference can be attributed to the likelihood of algal blooms covering the entire extent of medium to small-sized lakes, while in the case of larger lakes (such as Lakes Ontario, Victoria and Baikal), blooms were primarily concentrated in the shallow nearshore regions [34].

European lakes exhibited the most pronounced occurrences of algal blooms, with a median annual bloom frequency of 5.0%. This is more than twice the lowest bloom frequency observed in Oceana lakes. Remarkably, many lakes located in the Southern Alps of Europe and around the Black Sea region displayed annual bloom frequencies exceeding 10%. North America ranked second in median annual bloom frequency (3.7%) after Europe. Lakes with more frequent blooms were primarily concentrated in Central North America, encompassing the Mississippi River basin and the Lake Winnipeg watershed. These regions are dominated by agricultural areas, characterized by substantial nutrient influx from animal waste and fertilizer application, contributing to the heightened prevalence of algal blooms [35]. In Asian lakes, high-incidence regions of algal blooms were primarily located in Central and East Asia, particularly in the lower Yangtze River Basin. In the Southern Hemisphere, frequent algal blooms were observed in lakes situated in southeastern South America, and eastern and southern Africa. However, the overall bloom frequency in the Southern Hemisphere was lower compared to the Northern Hemisphere.

The global distributions of bloom onset and bloom persistence exhibit distinct latitudinal dependencies (Fig. S2). In tropical regions, blooms can persist throughout the entire year. As we move towards higher latitudes in the Northern Hemisphere, the onset of blooms progressively shifts to later months. In these high-latitude lakes, blooms are frequently observed during the summer months, with a bloom persistence lasting <3 months.

### Pervasive increasing trends

**Figure 2.**
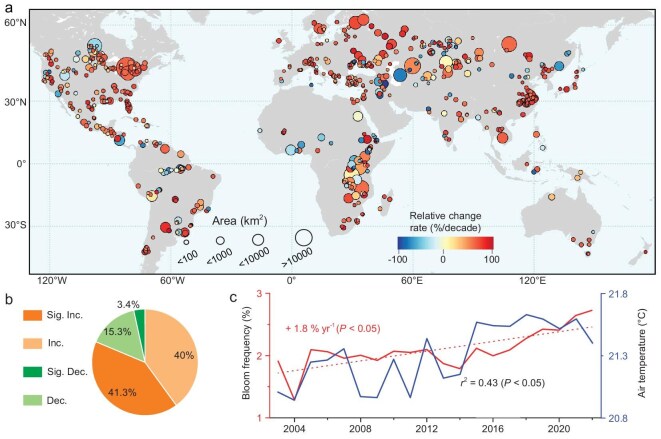
Global trend of algal blooms in large freshwater lakes between 2003 and 2022. (a) Linear slopes of the bloom frequency for lakes that exhibited blooms for at least 10 years during our study period (*n* = 620). (b) The proportions of four distinct trends: significant increase (Sig. Inc.), non-significant increase (Inc.), significant decrease (Sig. Dec.), and non-significant decrease (Dec.). (c) Long-term changes in global median bloom frequency for bloom-affected lakes over the study period, with the linear slope and *P*-value annotated. Also plotted are the corresponding median air temperatures concurrent to bloom events, and with the correlation to bloom frequency indicated. The colors of the curves correspond with the y-axis.

Of the 620 large lakes that exhibited blooms for at least 10 years during our study period, we observed an upward trend in bloom frequency in 504 lakes (Fig. 2). Furthermore, we identified statistically significant increases (*P* < 0.05) in 256 lakes. In contrast, decreasing trends were observed in 116 lakes, and only 3.4% of these exhibited statistically significant decreases. As a result, the global median annual bloom frequency for all bloom-affected lakes indicated a significant (*P* < 0.05) annual relative increase of +1.8%/yr.

Although the surge in algal bloom frequency is evident across our globally distributed study sites, our findings indicated that these changes manifest in distinct temporal and spatial patterns. Notably, the relative increase in annual bloom frequency was particularly pronounced in subtropical regions (+2.3%/yr, *n* = 189) (Fig. S3). In these regions, 51.9% of lakes showed significant increases, while only 11.1% displayed decreasing trends. In comparison, tropical zones displayed a relatively weaker increasing trend (+1.4%/yr, *n* = 150), with the highest proportion of lakes exhibiting decreasing trends (29.3%). The global increase in bloom frequency in lakes primarily occurred after 2015. When comparing the median relative growth rates between the periods of 2003–2015 and 2016–2022, the latter (+4.6%/yr) displayed a fourfold rate of acceleration compared to the former period (+1.1%/yr) (Fig. S4A). These findings align with the results of Hou *et al.* [2], which indicated that the global increase in algal blooms was predominantly observed during the 2010s, based on decadal mean values.

Further analysis reveals that among the lakes with higher bloom frequency during 2003–2015, their rates of increase during the 2016–2022 period are notably higher than those of lakes with comparatively less frequent blooms in the preceding period (matched pair t-test, *P* < 0.05, Fig. S4B). In other words, lakes experiencing more frequent algal blooms are deteriorating at a faster rate.

The worldwide increase in algal blooms is further corroborated by the widespread occurrence of earlier onset dates and prolonged bloom persistence (Fig. S2). Over the period from 2003 to 2022, the average onset of bloom-affected lakes has advanced at a rate of 0.9 ± 1.8 days/yr, while the duration of bloom persistence has increased at an average rate of 1.6 ± 2.9 days/yr. Geographically, the shifts in these two key parameters align closely with changes in bloom frequency, with the most pronounced changes observed in sub-tropical lakes.

### Important role of climate warming

Globally, the median annual bloom frequency of the 962 bloom-affected large lakes exhibited consistent fluctuation patterns corresponding to the median air temperatures recorded from 2003 to 2022, displaying a statistically significant correlation (*r*^2^ = 0.43, *P* < 0.05). Notably, the lowest air temperature in 2004 corresponded well with the minimum bloom frequency, while the substantial increase in bloom frequency after 2015 coincided with a marked rise in air temperature.

To further elucidate the influence of air temperature on algal blooms in individual lakes, we investigated the correlation between the daily bloom frequency, as captured by satellite imagery, and concurrent air temperature, alongside other meteorological factors (precipitation and wind speed, see Methods). The influence of air temperature [16], precipitation [20] and wind speed [36] as contributors to the lake algal blooms has been widely demonstrated in previous studies, and these impacts are manifested at short time scales (from hours to days). Furthermore, we examined the correlations between bloom frequency and the utilization of nitrogen (N) and phosphorus (P) from fertilizers within the drainage basin of the studied lakes. The objective was to discern whether either nutrient enrichment or climate warming plays the more important role in the observed increasing trend of algal blooms.

**Figure 3.**
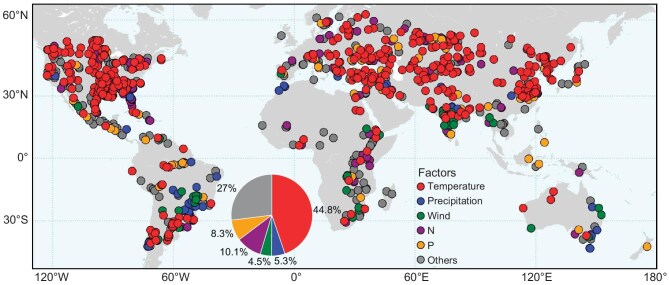
Correlations between bloom frequency and various potential factors contributing to bloom formation in lakes. The factors examined include air temperature, precipitation, wind speed, and local usage of nitrogen (N) and phosphorus (P) fertilizers. Each lake is denoted by a specific color, signifying the factor with the highest significant correlation (*P* < 0.05) to bloom frequency in the lake. The pie chart illustrates the proportions of different factors displaying the highest correlations. If bloom frequency does not exhibit a significant correlation with any of the factors, the lake is labeled as ‘others’.

Our results demonstrated a stronger correlation between daily air temperature and bloom frequency when compared to other meteorological parameters and nutrient concentrations, particularly in 44.8% of the bloom-affected lakes (Fig. 3). This strong correlation between bloom area and air temperature remained consistent across most lakes situated north of the Tropic of Cancer. In our comprehensive analysis of the entire daily dataset, we observed that 59.4% of the algal blooms occurred when the air temperature exceeded 20°C (Fig. S5A). These findings underscore the contribution of elevated temperatures to the increasing trend of lake algal blooms, especially considering that >97% of global lakes have experienced warming over the past decades [37]. The stimulating effects of warming on algal blooms are attributable to two factors: (1) bloom-forming phytoplankton species, such as cyanobacteria, have higher optimal growth rates compared to other algae [38,39]; (2) the warmer conditions offer extended growing seasons and create a more stratified water column, which promotes surface blooms [40]. In turn, recent changes in water quality, including algal blooms or browning [41], may also influence a lake's heat-trapping capacity. However, due to the lack of detailed data at this stage, it is difficult to quantify the interactions between these factors.

Our results show that the number of lakes with bloom frequency significantly correlated (*P* < 0.05) to either N or P usage was much less than those correlated to air temperature alone (Fig. S6). However, these results should not be interpreted as suggesting that nutrient availability is not important for bloom formation. In fact, further analysis revealed a significant correlation (*P* < 0.05) between mean annual total anthropogenic N inputs to the terrestrial biosphere and bloom frequency, contrasting with the weak correlation observed for atmospheric N. Significant correlations were also found for subsets of total anthropogenic N inputs, such as those from cropland, pasture, fertilizer or manure (Fig. S7), underscoring the role of human-induced nutrient enrichment in driving lake algal blooms.

In lakes where bloom frequency did not significantly correlate with nutrient enrichment, this could be because current saturated nutrient input levels may no longer be the primary limiting factor due to excessive nutrient inputs, internal nutrient recycling or legacy nutrient loading [42–44]. Another possible explanation is that algal blooms may result from the combined influence of various meteorological and nutrient factors, where a significant correlation with bloom frequency might not be apparent for any single factor alone. This aligns with the fact that 27% of the lakes in our study exhibit no significant correlation with either meteorological or nutrient factors (Fig. 3). These findings are consistent with an analysis of over 1000 US lakes, where nutrients had a significant impact on oligotrophic lakes, while in more eutrophic systems, temperature and its interactions with nutrients played a more prominent role [45]. Indeed, the responses of phytoplankton biomass to nutrients can exhibit various forms, such as log-linear, sigmoidal or other patterns, and these may vary among different bloom species and ecosystems [46,47].

It is also important to note that while the correlation between nutrient levels and temperature with algal bloom frequency indicate statistical relationships, they provide only a partial explanation of causality when considering the complex biological characteristics of algae [48]. Additionally, while we acknowledge that, in addition to fertilizer application, wastewater discharge, inland aquaculture, and livestock operations are significant nutrient sources in aquatic ecosystems, global data on these sources are often difficult to obtain due to technical and standardization challenges, or are associated with considerable uncertainties [49]. Importantly, our study does not seek to downplay the role of nutrient sources in algal growth. Rather, it aims to highlight the amplifying effect of global warming on algal blooms by revealing the significant relationship between warming and the increasing trend in algal blooms. Building on previous research on the impacts of warming on reproduction rates [18], stratification [40] and the length of the growing season [50], we emphasize the critical role of warming in the recent intensification of global lake algal blooms.

Lakes with relatively weaker correlations with air temperature are predominantly located in tropical regions (Fig. 3 and Fig. S6). In these lakes, temperature is less likely to act as a factor controlling bloom occurrences [51]. Nevertheless, our observations also identified ∼24.6% of the blooms occurred at temperatures ≤16°C (Fig. S5A). This finding supports a recent study suggesting that algal blooms can also form under relatively low temperatures [52]. Our results also confirmed the decreased likelihood of algal blooms under conditions of high precipitation or strong winds (Fig. S5B). These meteorological conditions tend to vertically mix phytoplankton through the water column, preventing the observation of surface algal blooms from satellites [53,54]. However, when examining long-term trends on a global scale, the impact of wind speed or precipitation on bloom frequency was considerably smaller when compared to temperature.

Our analysis offers an unprecedented detailed temporal representation of algal blooms in global large lakes over the last two decades. While previous global-scale studies relied mainly on 30-m resolution Landsat data [2,25,55], which provided coverage for more lakes but much lower observation frequency, our approach allows for a more comprehensive understanding of the dynamic characteristics of algal blooms. We not only reaffirm the marked surge in such blooms since the 2010s (with a more precise identification of the increase after 2015) [2] but also highlight the important role of global warming in driving this trend. Notably, a recent study on coastal oceans has similarly revealed the stimulating effect of warming on phytoplankton blooms, particularly in mid-to-higher latitudes such as the Baltic Sea and the Alaskan coast [1], further emphasizing the wide-reaching consequences of warming on global aquatic ecosystems. It is important to recognize that while our study focused on large lakes (>50 km^2^) due to satellite spatial resolution constraints, smaller lakes are also witnessing a similar increase in algal blooms [56,57], and their greater numbers warrant similar consideration in future research efforts. Additionally, optical remote sensing is limited by cloud cover interference, with a global average cloud-free coverage of ∼55% over land and marked spatiotemporal variability [58]. To mitigate this constraint, we utilized daily satellite observations and implemented a cloud-cover weighted bloom frequency estimation method (see Methods).

Our mapped lake algal bloom dataset serves multiple important purposes. It not only provides valuable information for a more detailed analysis of the recent increase in algal blooms and its connection to environmental degradation, such as oxygen depletion [59] and biodiversity losses [60], but also lays the foundation for assessing the broader impacts of eutrophication on ecosystems and socio-economics (e.g. fisheries, tourism, drinking water supplies) at regional or global scales [4]. Our daily monitoring of the lake bloom patterns, combined with process-based models for nutrient transport, holds the potential to deepen our understanding of the mechanisms governing algal bloom onset and progression, and it can contribute to the development of more robust strategies for mitigating such incidents. All these efforts align with the United Nations’ sustainable development goals, particularly those related to human health, water security and biodiversity conservation [61].

## MATERIALS AND METHODS

### Data sources

#### Satellite images

We used the complete archive of images captured by the Moderate Resolution Imaging Spectroradiometer (MODIS) aboard the Aqua satellite during the period from 2003 to 2022. The MODIS level-1A images were obtained from the U.S. National Aeronautics and Space Administration (NASA) Goddard Space Flight Center (GSFC). The MODIS instrument captured images for the entire globe with a spatial resolution of 1–2 days, making it the most extensive and consistent source of daily observations. A total of 0.8 million images were used in our study. The MODIS images were processed using SeaDAS software (Version 7.6) to derive Rayleigh-corrected reflectance (Rrc, dimensionless) [62], which is a satellite product that has been widely used for the detection of algal blooms or other pelagic features [23,24]. All MODIS Rrc images were then mosaicked into global daily Rrc composites for further detection of algal blooms, and this process was also accomplished using the l3_gen module in the SeaDAS. Note that, as Rrc data is not readily available as a NASA standard product, and other atmospherically corrected products (such as surface reflectance products) on water surfaces suffer from severe patchiness issues and other problems [63,64], global processing of Rrc and its subsequent use in algal bloom detection is currently unavailable.

#### Meteorological dataset

We acquired daily meteorological data from the European Centre for Medium-Range Weather Forecasts (ECMWF) Re-Analysis v5-Land dataset (ERA5-Land) [65]. ERA5-Land offers data spanning from 1981 to the present day, with a spatial resolution of 0.1° × 0.1°. The variables used in our analysis include 2-m surface air temperature, wind speed and precipitation, which were considered important parameters influencing lake algal blooms [13,14]. Given the 0.1° × 0.1° (∼10 km × 10 km at the equator) spatial resolution of the meteorological data, the central pixel of a lake generally represents the overall meteorological conditions of the respective lake area. A comparison of concurrent water [37] and air temperatures shows that, while lake water temperature changes typically lag behind fluctuations in air temperature, surface water generally responds to air temperature within the same day. Due to the unavailability of complete water temperature data for all the examined lakes, we have used air temperature as a proxy for lake water temperature.

#### Nutrient data

We obtained a global gridded dataset (0.5° × 0.5°) for synthetic nitrogen (N) and phosphorus (P) fertilizers usage in croplands. This dataset, spanning the years 1900 to 2013, was produced by Lu and Tian [66]. We summarized the annual N and P fertilizer usage within each lake basin, where the basin boundaries were derived from the HydroBASINS dataset (https://www.hydrosheds.org/page/hydrobasins/). A global dataset of annual anthropogenic N inputs at a 5-arcmin resolution, produced by Tian *et al.* [67], was applied to lakes for the period from 2003 to 2019. We also obtained the global and national total N and P fertilizer usage data from 2003 to 2020, which were compiled by the Food and Agriculture Organization (FAO) (https://ourworldindata.org/fertilizers).

### Global algal bloom detection and trend analysis

The method employed to extract areas affected by algal blooms was recently developed by Wang *et al.* [28]. A brief summary of the primary processing steps is as follows: A pivotal step involves employing a thresholding scheme to distinguish pixels containing algal blooms from those that are bloom-free. This differentiation is achieved using a normalized Floating Algal Index (nFAI), derived from MODIS Rrc data at the bands of 555, 645, 859 and 1240 nm [62]. Subsequently, chromatic coordinates within the CIE colorimetry space were determined, using MODIS red (645 nm), green (555 nm) and blue (443 nm) bands. These coordinates serve the purpose of masking non-green features, such as clouds and cloud shadows. To further refine the results, areas potentially influenced by floating-leaved vegetation were excluded. This was accomplished by identifying regions with lower open water presence, determined using historical water presence probability data provided by the Global Surface Water Occurrence (GSWO) dataset [68]. The rationale behind this step lies in the observation that regions with vegetation cover are expected to exhibit substantially lower water presence compared to areas affected by algal blooms. We note that, although Wang *et al.* [28] primarily focused on analyzing lakes in China, their methodology was developed and rigorously tested using a dataset that includes lakes from various parts of the world. Their comprehensive validation process showcased the reliability of the algorithm, achieving consistently high accuracy levels of 81%.

We conducted algal bloom extraction for all freshwater lakes and reservoirs (collectively referred to as ‘lakes’ in our study) worldwide with a size exceeding 50 km^2^, and the boundaries of these lakes were obtained from the GLAKES dataset [29]. While the global count of such large lakes is 3646, some of them exhibit elongated shapes, which can introduce substantial mixing-pixel issues in remote sensing observations. Therefore, our investigation concentrated on a subset of 1956 lakes distributed worldwide (see Fig. 1), and these lakes collectively represent 51% of the total surface area of freshwater lakes on Earth. Note that, although algal blooms have also been detected in some endorheic saline and brackish lakes [69], in certain other endorheic lakes with clear water, they may appear green due to high mineral content [2,70]. This can introduce large errors to our algorithm (based on the CIE colorimetry space) and, as a result, these lakes have been excluded from our analysis using the boundaries of endorheic basin [71]. Generally, such lakes are predominantly found in mountainous or desert regions, experience limited nutrient enrichment, and have fewer issues with algal blooms at the global scale [71].

We performed algal bloom extraction over all daily global Rrc composites between 2003 and 2022. For lakes where blooms were identified during the last two decades (*n* = 962, namely bloom-affected lakes), we derived three parameters characterizing algal blooms lake algal blooms, including frequency, onset and persistence (Fig. S1).

For each daily observation, the bloom frequency was quantified as follows:


(1) \begin{eqnarray*}
{\mathrm{Bloom\,\,{\rm frequency}}} = \frac{{{A_{\mathit{Bloom}}}}}{{{A_{\mathit{valid}}}}}{\left( {\frac{{{A_{\mathit{valid}}}}}{{{A_{\mathit{Lake}}}}}} \right)^2},
\end{eqnarray*}

where *A_Bloom_* is the satellite-detected algal bloom area, *A_valid_* represents the valid satellite observational area within the lake and *A_Lake_* is the lake surface area. The use of (*A_valid_/A_Lake_*)^2^ as a weighting factor aims to increase the weighting of observations with lower cloud cover in the time series, thereby enhancing our confidence in results derived from higher-quality data. This approach is consistent with the methodology employed by Wang *et al.* in the detection of lake ice phenology within a year [72].

Bloom onset represented the first day of the year when the initial bloom was detected, while bloom persistence measured the duration between the first and last bloom observations within that year. To address seasonal differences between the Northern and Southern Hemispheres, we incorporated ice phenology data, which also helped mitigate misinterpretations of remote sensing algal blooms due to variability in ice reflectance influenced by factors like ice thickness, morphology and satellite observation angles [2]. For lakes with winter ice cover, we calculated the average ice cover period for each lake using data from Wang *et al.* [72]. Bloom onset for these lakes was determined as the first bloom detected after ice melt in spring, and bloom persistence was the duration from onset to the last observation before winter freeze. This method effectively addressed hemispherical seasonal differences for frozen lakes in the Southern Hemisphere. For the 91 non-frozen lakes in the Southern Hemisphere, bloom onset was adjusted to occur 6 months later to reflect hemispherical seasonal differences. Notably, bloom persistence does not indicate the number of consecutive days when algal blooms continuously occur. Instead, it represents the period during which algal blooms have the potential to occur within a year, given suitable meteorological and hydrological conditions. To further facilitate our determination of bloom onset and persistence, we applied a 15-day running mean to smooth the daily bloom frequency within each year (Fig. S1). Then we calculated the annual bloom frequency as the median value of smoothed daily bloom frequencies within the bloom persistence period.

For lakes with blooms that occurred in more than half of the observed years, we analyzed their trends in bloom frequency, onset and persistence, where the change rates were estimated using linear regression slopes through their annual values.

### Correlation analysis with potential driving factors

To discern the potential drivers behind the recent upsurge in global algal blooms, we conducted correlation analyses between bloom frequency and various factors, including nutrient levels and meteorological conditions (Fig. 3 and Fig. S6). Since the formation and disappearance of surface blooms are highly sensitive to episodic meteorological conditions (ranging from hours to days) [6,73], we examined the correlations between daily bloom frequency and daily mean air temperature, wind speed and precipitation for all 962 bloom-affected lakes. Furthermore, we conducted a correlation analysis between annual bloom frequency and the corresponding usage of N and P fertilizers within the lake basins. This analysis aimed to investigate whether interannual variations in nutrient enrichment could influence algal blooms in these lakes. The rationale for conducting separate temporal analyses of nutrients and meteorological factors is that short-term fluctuations in nutrient concentrations are less likely to exert as significant an instantaneous impact on bloom growth as meteorological factors [74]. Additionally, the acquisition of nutrient data at a daily scale is challenging [75,76]. Furthermore, since global-scale grid data on fertilizer usage was only available to 2013 [66], we utilized FAO national-level statistical data to analyze the potential link between nutrient usage and interannual variations in algal blooms (Table S1). We acknowledge that the nutrient status of lakes is influenced by various local factors, including soil type, land use, rainfall, hydrology (i.e. river network and flow conditions), sewage treatment, etc. [77,78]. However, quantifying the impact of each of these factors for every lake is challenging and beyond the scope of this study. Furthermore, the precipitation data employed in this study were extracted from the lake center points rather than encompassing the entire watershed scale. This approach was undertaken to analyze the influence of short-term rainfall events on lake hydrodynamic disturbances and subsequent algal blooms [79,80]. In contrast, precipitation analysis at the watershed scale further reflects the impact on nutrient transport within the watershed [81], which has already been accounted for through fertilizer usage data.

To further incorporate analysis on anthropogenic N inputs, we utilized an additional dataset that spans N inputs from fertilizers, manure and atmospheric deposition [67]. This dataset, which covers the period from 2003 to 2019, accounts for N inputs across different land use types and N forms, resulting in 10 distinct N input categories: (i) NH_4_^+^-N fertilizer applied to cropland, (ii) NO_3_^−^-N fertilizer applied to cropland, (iii) NH_4_^+^-N fertilizer applied to pasture, (iv) NO_3_^−^-N fertilizer applied to pasture, (v) manure N application on cropland, (vi) manure N application on pasture, (vii) manure N deposition on pasture, (viii) manure N deposition on rangeland, (ix) NH_x_-N deposition and (x) NO_y_-N deposition. We then calculated the annual relationships between bloom frequency and total N inputs, as well as specific subsets such as N fertilizer (i + ii + iii + iv), manure N (v + vi + vii + viii), atmospheric N (ix + x), cropland (i + ii + v) and pasture (iii + iv + vi + vii) N inputs. It is also important to note that the annual average meteorological conditions may obscure the short-term response of algal blooms to these meteorological conditions, and we did not include an annual correlation analysis here.

## Supplementary Material

nwaf011_Supplemental_File

## DATA AND MATERIALS AVAILABILITIES

The satellite-based dataset of global freshwater lake algal blooms can be found at https://zenodo.org/records/14799491.
