# Supplementary material for: Global elevation of algal bloom frequency in large lakes over the past two decades
Source: Natl Sci Rev. 2025 Jan 11;12(3):nwaf011. doi: 10.1093/nsr/nwaf011 (PMC12089762; doi:10.1093/nsr/nwaf011)
Supplement: nwaf011_Supplemental_File [file nwaf011_supplemental_file.docx]

**Supplementary Materials**

**Supplementary Figures**


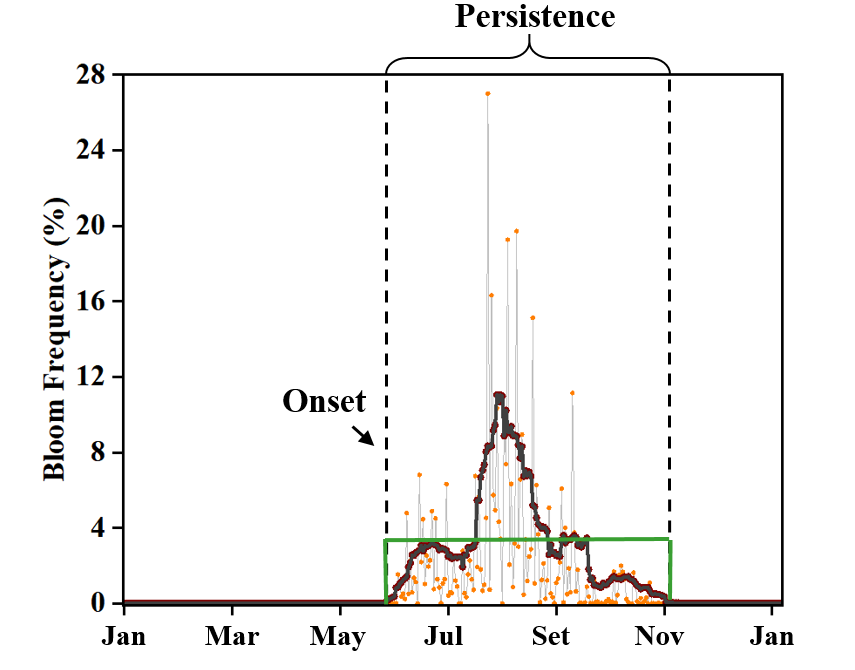


**Figure S1. An example of how the bloom onset and persistence are determined through satellite-detected annual time series of daily bloom frequencies for a lake located in the Northern Hemisphere.** The orange and black data points represent the original and 15-day running mean smoothed daily bloom frequencies, respectively.


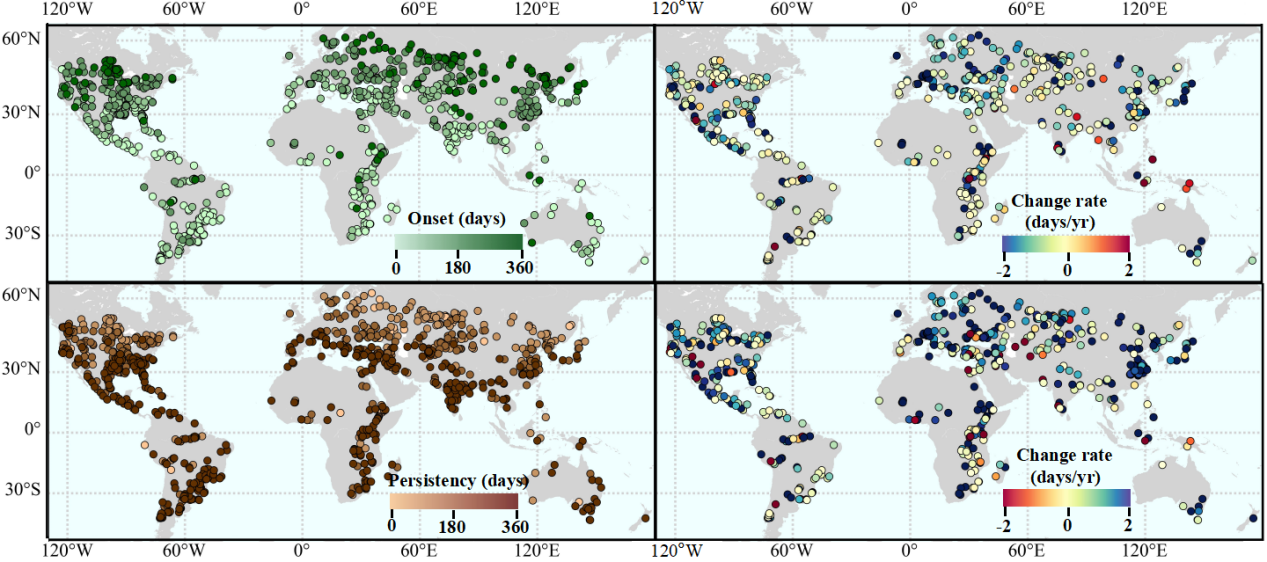


**Figure S2. Global patterns (left panels) and trends (right panels) in bloom onset (upper panels) and bloom persistence (lower panels).**


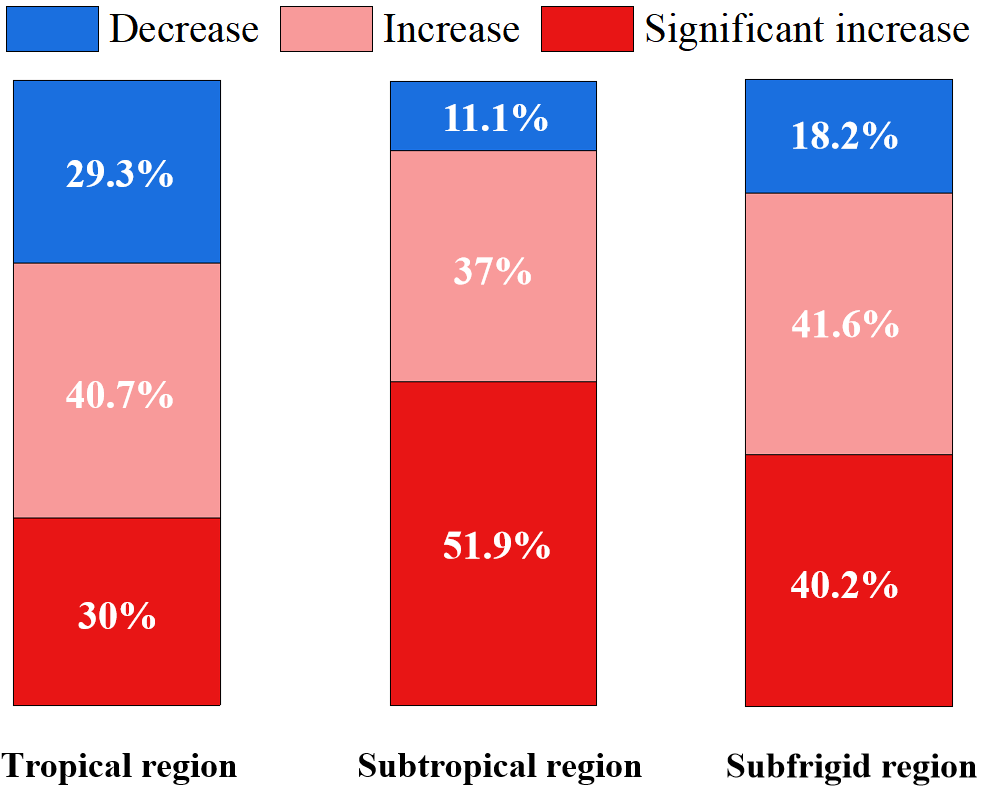


**Figure S3. Proportions of different trends of lake algal blooms in three distinct climatic zones: tropical (n=150), subtropical (n=189), and subfrigid (n=281).**


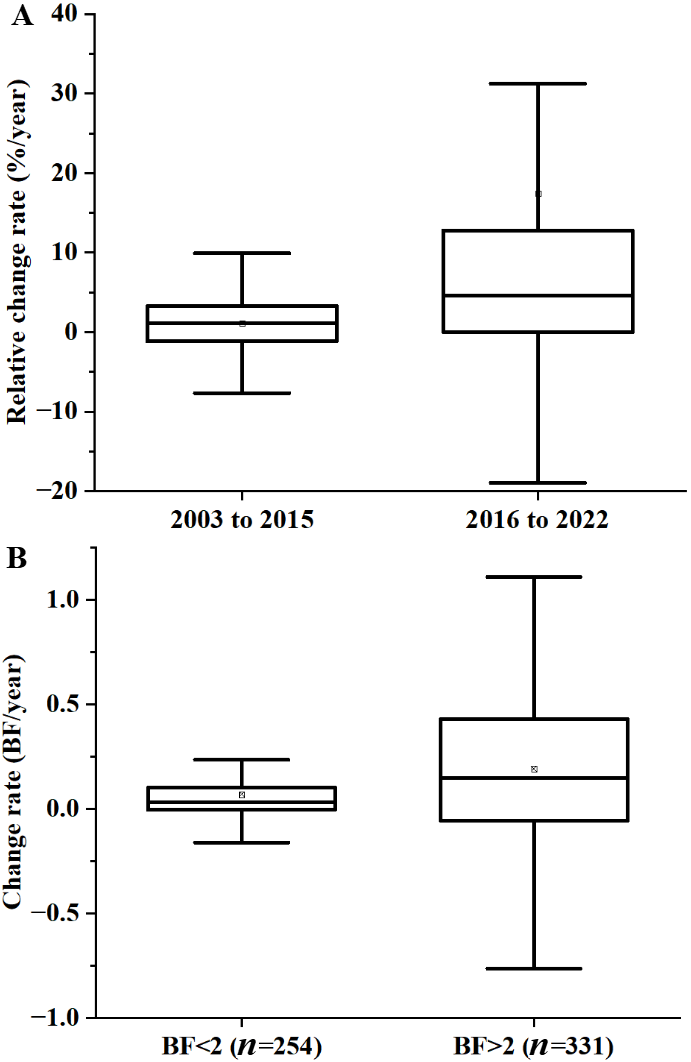


**Figure S4. Comparison of the bloom trends for different periods and lake groups. (A)** Comparison of the trends between two periods: 2003-2015 and 2016-2022. The boxplots show the relative change rates of annual bloom frequency for global bloom-affected lakes within different periods. (**B**) Comparison of the trends from 2016 to 2022 for lakes with severe and less severe blooms. The partition of the two groups was based on whether the mean annual bloom frequency was greater than or less than 2% between 2003 and 2015. The center line of the boxplots represents the median value, the small square point indicates the mean value, the bottom and top bounds of boxes are first and third quartiles, and the whiskers show a maximum of 1.5 times the interquartile range.


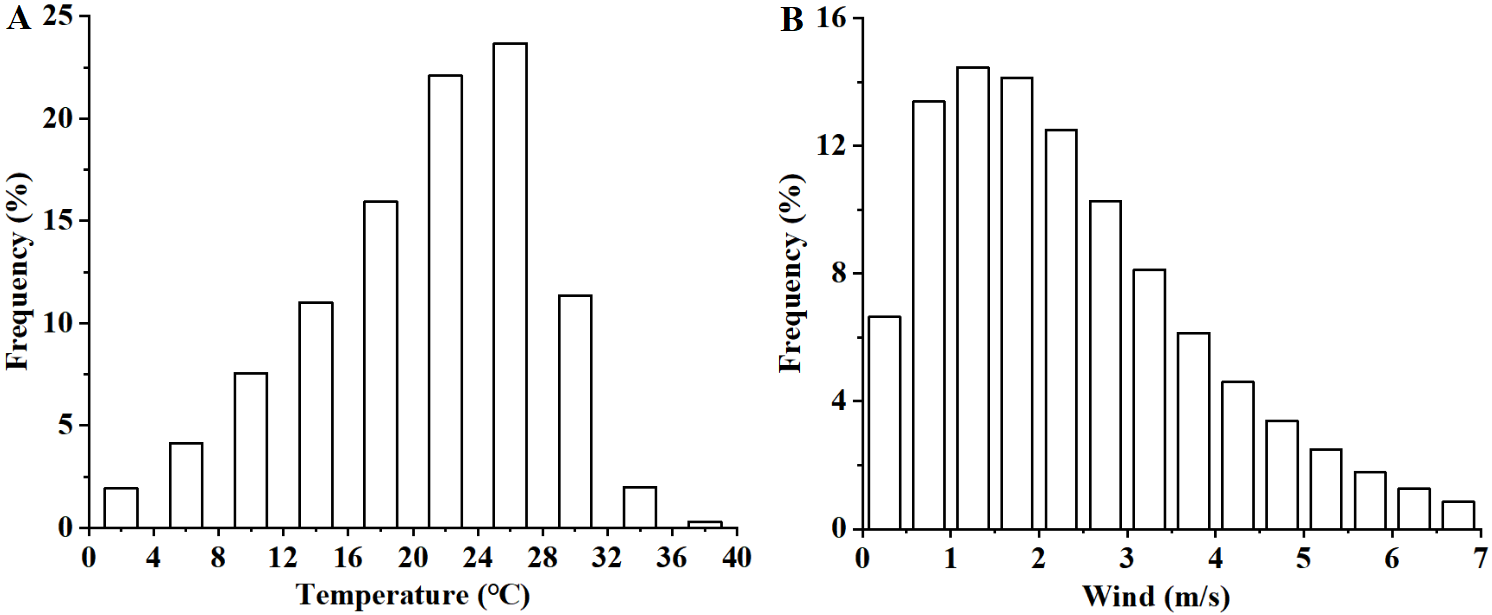


**Figure S5. Histogram distributions of daily air temperature and wind speed on days when algal blooms were observed.** The histograms were generated using all our historical observations from 2003 to 2022.


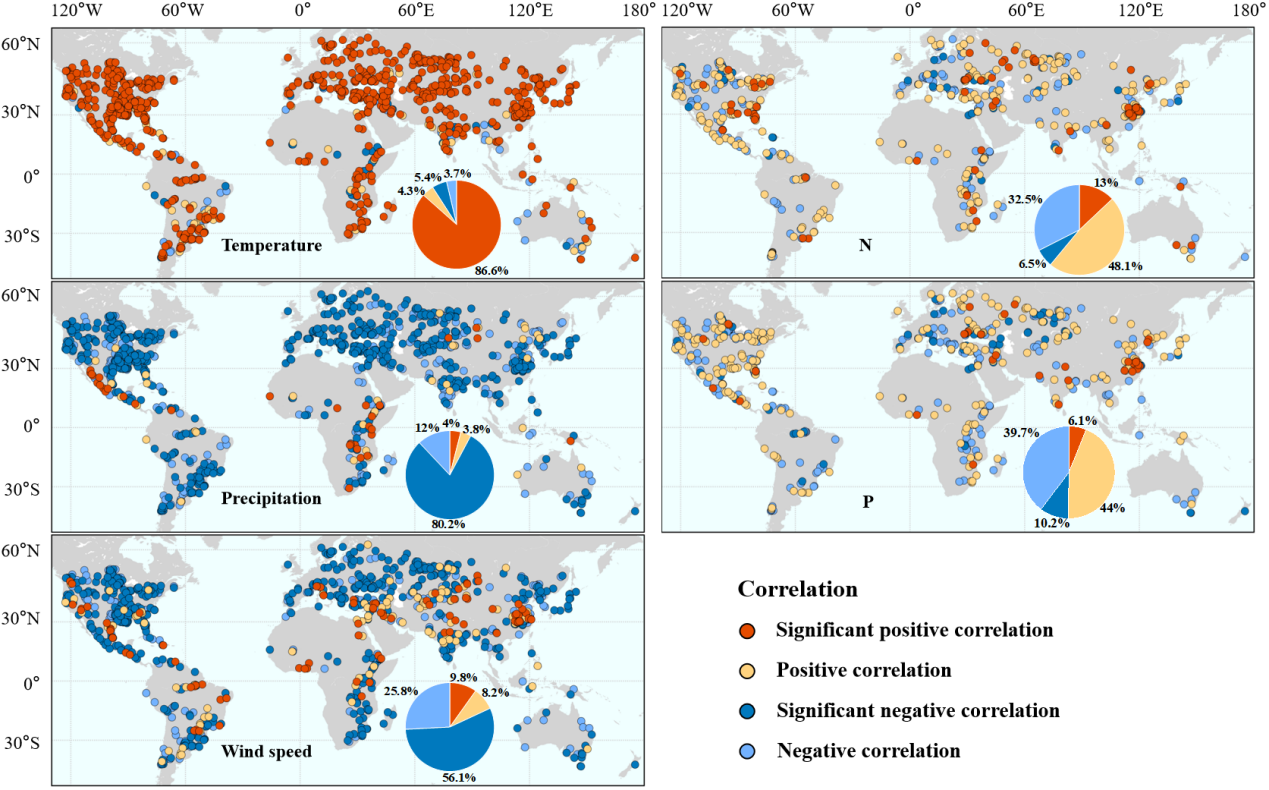


**Figure S6. Correlations between bloom frequency and various driving factors, including air temperature, wind speed, precipitation, and the usage of N and P fertilizers within the lake basins.** These correlations are categorized into four distinct types, and their fractions are demonstrated as a pie chart within each panel.


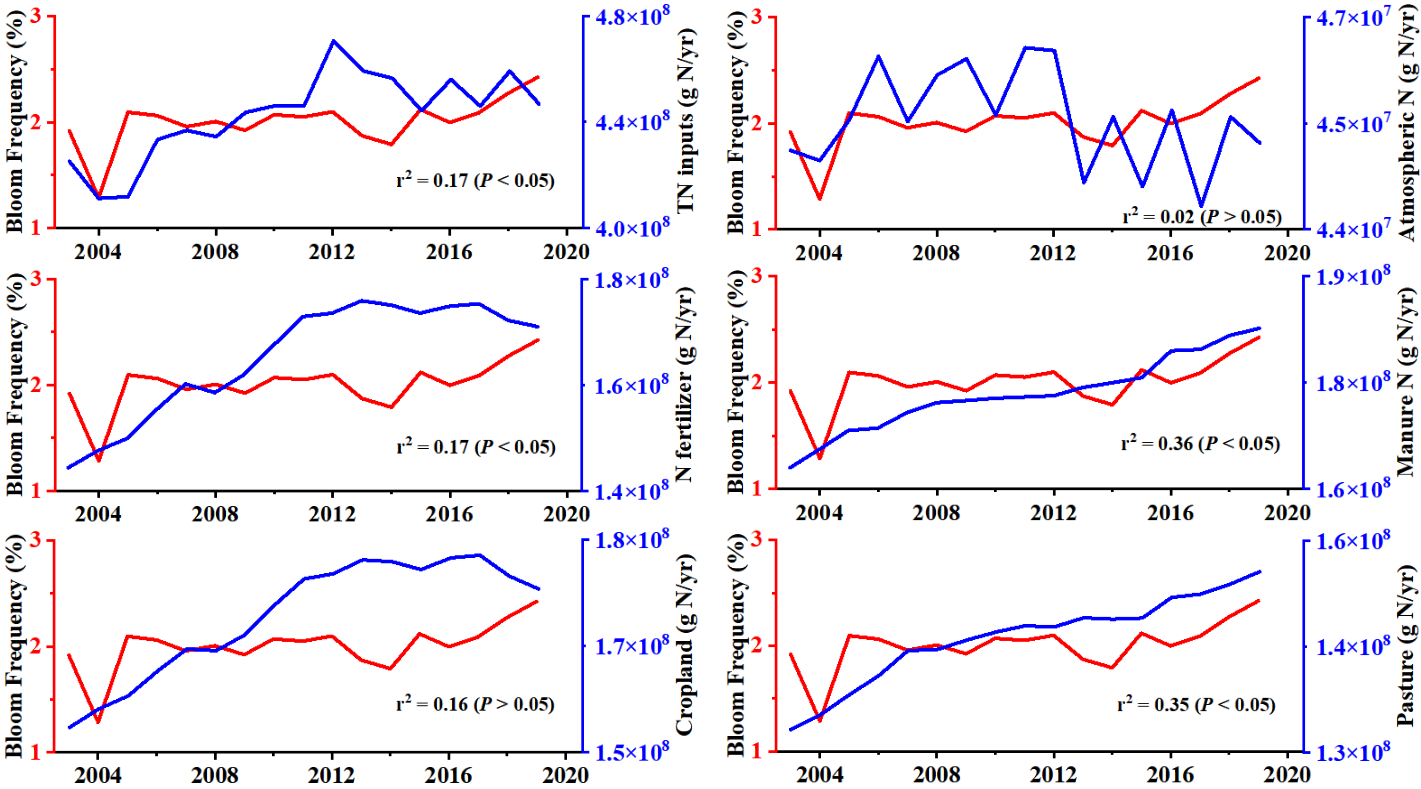


**Figure S7. Long-term trends (2003-2019) in various nitrogen inputs and their correlation with bloom frequency.**

**Table S1. Change rates of the usages of N and P fertilizers for different nations between 2003 and 2020.**

| **Continent** | **Country** | **Change rates of N (kg/yr)** | **Change rates of P (kg/yr)** |
| --- | --- | --- | --- |
| **Africa** | Algeria | 0.2867 | 0.2599 |
|  | Angola | 0.2292 | 0.0721 |
|  | Benin | 0.8559 | 0.4587 |
|  | Botswana | -1.7814 | 0.4426 |
|  | Burkina Faso | 0.2054 | 0.0608 |
|  | Burundi | 0.3818 | 0.4508 |
|  | Cameroon | 0.1742 | 0.0190 |
|  | Central African Republic | 0.0003 | -0.0007 |
|  | Congo | 0.0991 | 0.0480 |
|  | Egypt | -2.3936 | 1.0204 |
|  | Ethiopia | 1.2325 | 0.3723 |
|  | Gabon | 0.6726 | 0.4977 |
|  | Gambia | -0.2943 | -0.1307 |
|  | Ghana | 0.9384 | 0.6371 |
|  | Guinea | 0.0917 | 0.1150 |
|  | Ivory Coast | 0.2446 | 0.2855 |
|  | Kenya | 0.8174 | 0.6055 |
|  | Libya | -0.4782 | -0.1508 |
|  | Madagascar | 0.3853 | 0.0611 |
|  | Malawi | -0.0593 | -0.0695 |
|  | Mali | 0.7142 | 0.2651 |
|  | Mauritius | -0.7214 | -0.5507 |
|  | Morocco | -0.5405 | 0.1690 |
|  | Mozambique | 0.2184 | 0.0241 |
|  | Namibia | 1.2668 | 0.0522 |
|  | Niger | 0.0207 | -0.0056 |
|  | Nigeria | 0.4678 | 0.1543 |
|  | Rwanda | 0.4427 | 0.5332 |
|  | Senegal | 0.6362 | 0.2093 |
|  | Seychelles | 2.4120 | 0.9094 |
|  | South Africa | 0.0371 | 0.4844 |
|  | Sudan | 0.7470 | -0.0287 |
|  | United Republic of Tanzania | 0.4086 | 0.1592 |
|  | Togo | 0.1218 | 0.0598 |
|  | Tunisia | 0.2024 | 0.1406 |
|  | Uganda | 0.0307 | 0.0133 |
|  | Zambia | 2.2832 | 0.6692 |
|  | Zimbabwe | -0.1863 | 0.1377 |
| **Asia** | Afghanistan | 0.4831 | 0.1137 |
|  | Armenia | 11.0420 | 0.0250 |
|  | Azerbaijan | 3.6456 | 0.0480 |
|  | Bahrain | 0.8484 | -0.0344 |
|  | Bangladesh | 2.2571 | 4.0885 |
|  | Bhutan | 0.2698 | 0.1106 |
|  | Brunei | -0.0717 | 0.4554 |
|  | Cambodia | 1.8034 | 0.4072 |
|  | China | 0.6757 | 0.0892 |
|  | Cyprus | 1.0911 | 0.6242 |
|  | Fiji | 0.1367 | 0.5562 |
|  | Gaza Strip | -1.8915 | -1.1978 |
|  | Georgia | 1.6678 | 0.9011 |
|  | Indonesia | 0.3199 | 0.6902 |
|  | Iran | -1.9587 | -1.4302 |
|  | Iraq | 0.2859 | 0.2765 |
|  | Israel | -1.8915 | -1.1978 |
|  | Japan | -2.3298 | -3.6801 |
|  | Jordan | 3.3015 | -1.4232 |
|  | Kazakhstan | 0.1454 | 0.0832 |
|  | Republic of Korea | -4.8635 | 0.7806 |
|  | Kuwait | 9.3665 | 8.5642 |
|  | Kyrgyzstan | -0.3575 | 0.0424 |
|  | Lebanon | 0.6722 | 2.8947 |
|  | Malaysia | -2.9201 | -0.7393 |
|  | Maldives | 3.9580 | 0.0609 |
|  | Mongolia | 1.8330 | 0.0610 |
|  | Myanmar (Burma) | 1.2866 | 0.4883 |
|  | Nepal | 3.7690 | 1.6829 |
|  | New Caledonia | -1.6591 | -1.8180 |
|  | Oman | 0.1509 | 0.8171 |
|  | Pakistan | 1.5586 | 0.9745 |
|  | Papua New Guinea | 0.9487 | 0.0108 |
|  | Philippines | 0.6639 | 0.2010 |
|  | Saudi Arabia | -0.5253 | -0.5945 |
|  | Sri Lanka | -1.5710 | -0.3251 |
|  | Syria | -3.3937 | -1.5410 |
|  | Tajikistan | 1.2466 | -0.2953 |
|  | Thailand | 0.4546 | -0.2632 |
|  | Turkey | 1.7976 | 0.6050 |
|  | United Arab Emirates | 2.6484 | 2.0617 |
|  | Uzbekistan | 3.8852 | 1.7889 |
|  | Vietnam | 1.2778 | -0.9004 |
|  | Yemen | -0.0350 | 0.0309 |
|  | India | 2.5193 | 1.0072 |
| **Europe** | Albania | 0.0228 | 0.0723 |
|  | Austria | 0.7869 | -0.4507 |
|  | Belgium | 2.3236 | -2.2343 |
|  | Bosnia and Herzegovina | 3.6960 | 0.0199 |
|  | Bulgaria | 2.2234 | -0.3843 |
|  | Byelarus | -0.0107 | -0.6816 |
|  | Croatia | -5.5197 | -3.2805 |
|  | Czech Republic | 4.5345 | 0.3244 |
|  | Denmark | 0.9522 | -0.1267 |
|  | Estonia | 1.1312 | 0.0874 |
|  | Finland | -1.4159 | -0.5399 |
|  | France | -0.2244 | -0.5646 |
|  | Germany | -1.7572 | -0.2882 |
|  | Greece | 0.2062 | -0.7955 |
|  | Hungary | 2.6462 | 0.8199 |
|  | Iceland | 0.5928 | -0.9216 |
|  | Ireland | 1.4262 | 1.5844 |
|  | Italy | -1.0138 | -0.6258 |
|  | Latvia | 1.9857 | 0.6376 |
|  | Lithuania | 0.7549 | 0.3980 |
|  | Luxembourg | -1.2246 | -0.9982 |
|  | Macedonia | 0.9483 | 0.2072 |
|  | Malta | 7.3526 | 0.0446 |
|  | Moldova | 1.5171 | 0.8435 |
|  | Netherlands | -3.1514 | -2.2072 |
|  | Norway | 0.7254 | -0.5034 |
|  | Poland | 1.0651 | -0.0499 |
|  | Portugal | 1.3037 | -0.1198 |
|  | Romania | 1.3488 | 0.5683 |
|  | Russia | 0.4239 | 0.1495 |
|  | Slovakia | 2.7383 | 0.5104 |
|  | Slovenia | -2.1475 | -2.1281 |
|  | Spain | 0.4316 | -0.1485 |
|  | Sweden | 0.8976 | 0.0015 |
|  | Switzerland | -0.5759 | -0.4091 |
|  | Ukraine | 2.2278 | 0.5800 |
|  | United Kingdom | -0.9951 | -0.8904 |
| **North America** | Antigua and Barbuda | 0.0800 | -0.0738 |
|  | Bahamas | 13.8465 | 0.7920 |
|  | Barbados | -4.5509 | -0.4545 |
|  | Belize | 4.9950 | 5.2618 |
|  | Canada | 2.1626 | 0.9090 |
|  | Costa Rica | 1.9062 | -1.4154 |
|  | Cuba | 0.4527 | -0.0002 |
|  | Dominica | -0.7365 | -0.7179 |
|  | Dominican Republic | 2.2594 | 1.3060 |
|  | El Salvador | -1.3100 | -0.2544 |
|  | Guatemala | 2.8002 | 0.5231 |
|  | Honduras | 2.2285 | 0.9503 |
|  | Jamaica | -0.9044 | -0.3162 |
|  | Mexico | 1.5092 | 0.7622 |
|  | Nicaragua | 1.2817 | 0.2774 |
|  | Panama | -0.3050 | 0.7200 |
|  | St. Lucia | 0.1684 | -0.1249 |
|  | United States | 0.4500 | 0.0869 |
| **Oceania** | Australia | 0.6183 | -0.7387 |
|  | French Polynesia | -0.1486 | -0.2823 |
|  | New Zealand | 0.4890 | -1.0734 |
|  | Tonga | 0.1675 | 0.1355 |
|  | Western Samoa | -0.0033 | -0.0069 |
| **South America** | Argentina | 0.7971 | 0.3797 |
|  | Bolivia | 0.1565 | 0.1290 |
|  | Brazil | 2.6370 | 2.1779 |
|  | Chile | -0.9440 | 0.1093 |
|  | Colombia | -2.2552 | -0.9805 |
|  | Ecuador | 2.3964 | 0.5569 |
|  | Guyana | 0.9549 | 0.3058 |
|  | Paraguay | 1.0448 | 1.1265 |
|  | Peru | 0.9487 | 0.2110 |
|  | Suriname | 0.4093 | -0.2897 |
|  | Trinidad and Tobago | 3.2462 | 0.2271 |
|  | Uruguay | 2.2180 | 0.1577 |
|  | Venezuela | -0.9360 | 1.0599 |
